# The Use of Municipal Solid Waste Incineration Ash in Various Building Materials: A Belgian Point of View

**DOI:** 10.3390/ma11010141

**Published:** 2018-01-16

**Authors:** Aneeta Mary Joseph, Ruben Snellings, Philip Van den Heede, Stijn Matthys, Nele De Belie

**Affiliations:** 1 Magnel Laboratory for Concrete Research, Department of Structural Engineering, Faculty of Engineering and Architecture, Ghent University, Tech Lane Ghent Science Park, Campus A, Technologiepark Zwijnaarde 904, B-9052 Ghent, Belgium; aneetamary.joseph@ugent.be (A.M.J.); philip.vandenheede@ugent.be (P.V.d.H.); stijn.matthys@ugent.be (S.M.); 2 Strategic Initiative Materials (SIM vzw), Project ASHCEM within the Program “MARES”, Tech Lane Ghent Science Park, Campus A, Technologiepark Zwijnaarde 935, B-9052 Ghent, Belgium; 3 Sustainable Materials Management, VITO, Boeretang 200, 2400 Mol, Belgium; ruben.snellings@vito.be

**Keywords:** MSWI bottom ash, beneficiation, supplementary cementitious materials (SCMs), ceramics, clinker production, alternate fuel and raw materials (AFR)

## Abstract

Huge amounts of waste are being generated, and even though the incineration process reduces the mass and volume of waste to a large extent, massive amounts of residues still remain. On average, out of 1.3 billion tons of municipal solid wastes generated per year, around 130 and 2.1 million tons are incinerated in the world and in Belgium, respectively. Around 400 kT of bottom ash residues are generated in Flanders, out of which only 102 kT are utilized here, and the rest is exported or landfilled due to non-conformity to environmental regulations. Landfilling makes the valuable resources in the residues unavailable and results in more primary raw materials being used, increasing mining and related hazards. Identifying and employing the right pre-treatment technique for the highest value application is the key to attaining a circular economy. We reviewed the present pre-treatment and utilization scenarios in Belgium, and the advancements in research around the world for realization of maximum utilization are reported in this paper. Uses of the material in the cement industry as a binder and cement raw meal replacement are identified as possible effective utilization options for large quantities of bottom ash. Pre-treatment techniques that could facilitate this use are also discussed. With all the research evidence available, there is now a need for combined efforts from incineration and the cement industry for technical and economic optimization of the process flow.

## 1. Introduction

In the present consumer society, mass production and consumption of goods leads to the generation of large quantities of municipal waste. Initially, little attention was paid to the environmental burden of mass consumerism, and municipal waste was destined for disposal in dumps. Since the 1960s, increasing societal concern about health and ecological risks have invoked the implementation of advanced waste management systems in developed countries. The introduction of legislation and public waste management agencies aims to reduce the impact of waste disposal and control emissions of pollutants to the environment. Implementation of waste policies is one of the key policies of the European Commission. Waste reduction, collection, separation of recyclable or compostable materials and disposal of the residue to (sanitary) landfill or incineration facilities have now become common practice in solid waste management in many developed, densely-populated areas. In this respect, the hierarchy of waste management, also called Lansink’s ladder of reduce-reuse-recycle adopted as the European Union (EU) waste hierarchy, can lead to better utilization of resources and reduction of waste. In addition, the circular economy policy adopted by the EU urges closing the material use loop by utilizing ‘wastes’ for suitable applications [1]. Urban waste generation has surged to 1.2 kg/cap/day and total generation to around 1.3 billion tonnes per year, out of which, around 15% is incinerated. The percentage incinerated is as high as 62% in industrialized countries [2,3,4,5,6].

The main types of wastes subjected to incineration are municipal wastes, non-hazardous industrial wastes, hazardous wastes, sewage sludge and clinical wastes. Mostly, all non-recyclable non-hazardous wastes (MSW, waste from paper and wood industry, etc.) are co-incinerated and constitute the majority of the volume. Further discussions are about the residues from this fraction. The incineration process generates heat, and this is recovered and utilized as such or converted to electrical energy. In some cases, incineration is used solely for disinfection and volume reduction. Waste gets reduced to 10–15% of its volume and 20–35% of its weight after incineration [7]. The fate of the residues depends mostly on their environmental quality. Bottom ash is usually classified as non-hazardous and can thus be considered for various applications such as in road construction as a subbase material. Finer fractions (mainly boiler and fly ashes) can be hazardous due to salts, heavy metals, biocides and organics. Fly ash accounts for about 1–3% of the total residues [6]. Until the 1980s, major concern went to the gaseous emissions from the incinerators. Emissions were monitored for particulate matter, acidic gases including HCl, HF, HBr, HI, SO_2_, NOx, NH_3_, greenhouse gases etc., heavy metals like Hg, Cd, Tl, As, Ni, Pb, etc., and organics like CO, VOCs, polychlorinated dibenzo-p-dioxins (PCDDs), polychlorinated dibenzofurans (PCDFs), etc. In response, improved flue gas treatment systems were implemented, and the focus shifted to the management of the solid incineration residues instead [8].

Landfilling is a ‘go to’ solution for wastes that do not have any other destination; however, it has many disadvantages. Maintaining an engineered sanitary landfill is a costly affair, and it is a dead end for all the valuable resources in it. Furthermore, its land requirement, as well as the secondary pollution possibilities like pollution of groundwater due to leaching and release of landfill gases like methane, which has a high global warming potential, make it the least favorable waste disposal method. In response, the European Union published a directive in 1999 that aims to ban landfilling for all but hazardous waste. Furthermore, countries such as the United States and New Zealand instituted a landfill tax to prevent the recyclable resources being landfilled [5].

A prime target for utilization of incineration residues is construction materials. Both in terms of market volumes and the required technical quality, there is clear compatibility. Currently, bottom ashes are mainly used as road subbase material and building sand and gravel alternatives. Additionally, in Belgium, this utilization is limited to 102 kT out of around 400 kT produced. The reasons for this limited utilization and options for improving this situation are discussed further. Substituting primary resources by secondary raw materials closes material cycles and reduces natural resource depletion, thus contributing to the realization of a circular economy. Furthermore, the embodied energy related to initial production of recycled materials is often lower than that of primary materials. In addition to the depletion of raw materials, the impact of resource mining needs to be taken into account [9]. Use of MSWI ash in building materials can be considered as a viable alternative to landfilling, given the past successes in recycling of different waste streams (e.g., fly ash, blast-furnace slag, phospho-gypsum, bauxite fines, etc., [10].

## 2. Solid Waste Management and Incineration Ash Recycling in Belgium

In Europe, the European commission has set waste legislation, mainly divided into five subcategories and organizes various action plans as guidance for sustainable waste management for its member states. It provided the revised waste framework directive in 2008 that focusses on recycling at least 70% construction waste by 2020. The circular economy is one of the main policies and action plans that focusses on reducing landfilling to a minimum [11]. Belgium is politically divided into three regions, Flanders, Wallonia and Brussels Capital Region (BCR), for administrative purposes, and there are significant differences between the waste management policies of these three regions. In Flanders, waste management is carried out and regulated by OVAM (de Openbare Vlaamse Afvalstoffenmaatschappij), the public waste agency of Flanders. Two agencies responsible for waste management in BCR are Bruxelles Environnement and Agence Bruxelles Propreté. Enforcing extended producer responsibility has been the main strategy employed to improve the recycling rates in BCR. In Wallonia, the waste management is regulated by Département du Sol et des Déchets-DSD (Soil and Waste Department) (Wallonia, Belgium). Out of five million tons of MSW generated in Belgium in 2010, 9% was contributed by the Brussels Capital Region, 60% by Flanders and 31% by Wallonia [12]. 

**Figure 1:**
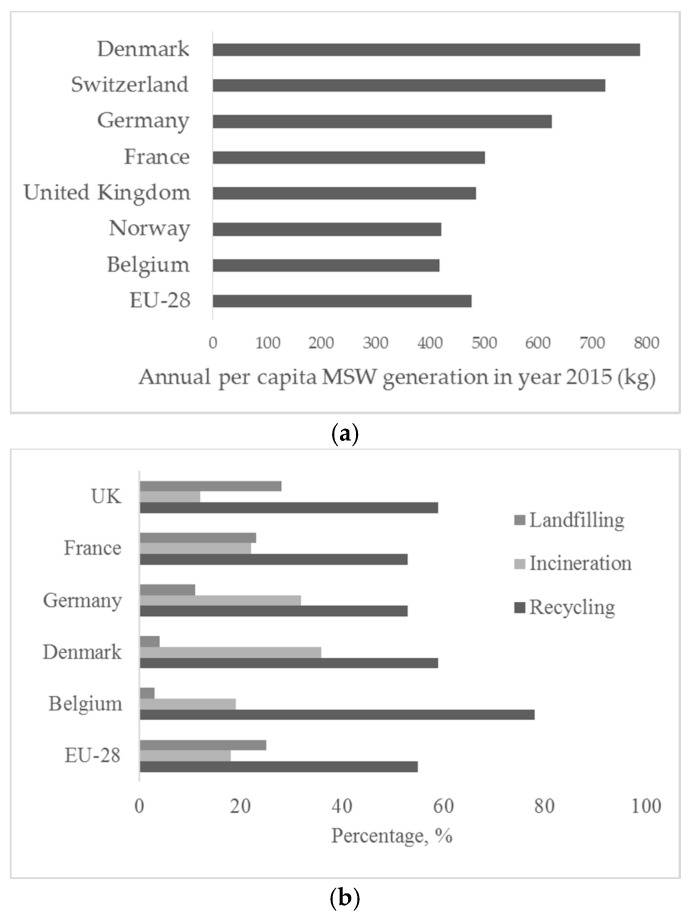
(**a**) Annual per capita generation of MSW in Belgium compared to the average value of EU nations and other prominent neighbors; (**b**) percentage of recycled, incinerated and landfilled waste cf. [13].

**Figure 2:**
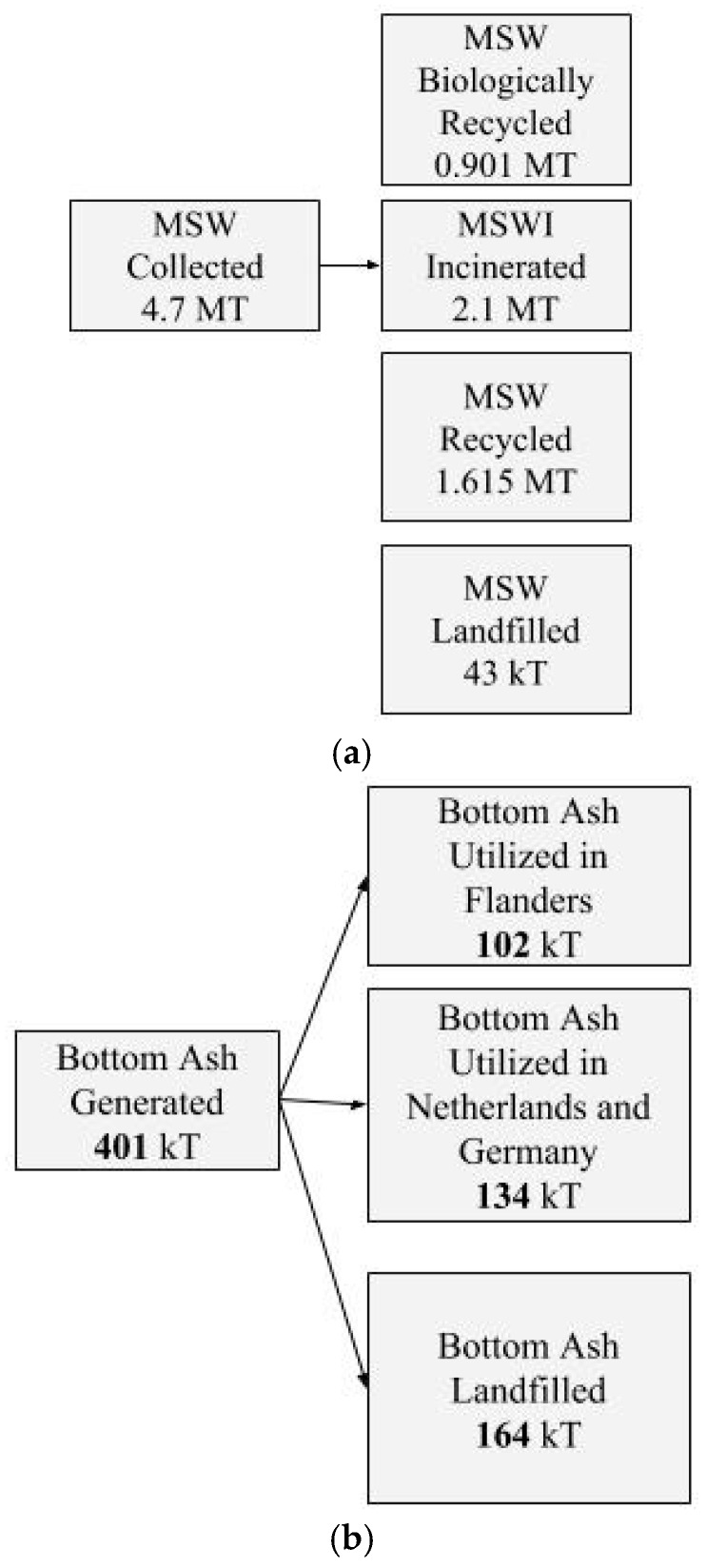
(**a**) Quantity of MSW generated and that composted, incinerated, recycled and landfilled in Belgium in 2015 [18]; (**b**) mass balance of bottom ash residue generated in Flanders in 2013 cf. [18].

Overall, Belgium is a front runner in achieving sustainability in waste management. It can be seen from Figure 1a that MSW generation is lower compared to the EU average. Figure 1b represents the comparison of waste treatment techniques in various countries. This includes other waste streams in addition to municipal solid wastes. Furthermore, the recycling percentage is much higher than average, while that of landfilled and incinerated waste is much lower than the EU-28 average. The quantities of municipal solid wastes that underwent different treatments are depicted in Figure 2a. Policy measures have been the major driver to increase the recycling level, in part by enforcing incineration and landfilling taxes, but also by defining environmental quality requirements that need to be met. 

In Flanders, the residues generated by incineration need to be treated to facilitate safe recycling as set out by VLAREMA (Vlaams reglement betreffende het duurzaam beheer van materiaalkringlopen en afvalstoffen), which is the Flemish implementation order of the sustainable materials management decree. The VLAREMA order describes the legal requirements (so-called end-of waste criteria) for waste materials to lose their “waste” status and become resources (obtain an end-of-waste certificate). VLAREMA specifically describes the environmental quality requirements for the use of recycled material as a construction material, by limiting the cumulative leached heavy metals at a liquid to solid ratio of 10, when tested according to the procedure in Compendium voor monsterneming en analyse CMA/II/A9.1. At present, these requirements are defined as contaminant leaching limits for metals and anions such as Cl and F and total concentrations for organic contaminants. In Wallonia, ‘Arrêté du Gouvernement wallon favorisant la valorisation de certains déchets’, published by the Walloon government, specifies the quality of bottom ash to be reused. It suggests to use the cleaned bottom ashes in shaped applications, and also, the environmental criteria limits with respect to leaching are more lenient compared to those of Flanders [14].

Two types of installations for bottom ash treatment are used in Europe, dry and wet installations. Dry installations give dry aggregates as end products with specific sizes. The processing consists of the following steps including cooling down the temperature of ash in air, primary ferrous metal separation, crushing, sieving, secondary ferrous separation, non-ferrous separation and ageing. Wet installations have an additional washing step to reduce the leachability of the final product, and the <2-mm fraction containing most contaminants is usually landfilled. However, the <2-mm portion constitutes around 50% of the total bottom ash generated. Incineration plants in Belgium adopt both wet and dry bottom ash treatment installations at different locations. At present, only waste incineration bottom ashes are treated in Belgium. The treatment processes adopted usually involve the recovery of ferrous metals by magnetic separation using over band magnets, which enables 55–60% ferrous metal recovery, mostly the coarser scrap. A ferrous metal content of 1.3–25.8% is typically left behind in the residue, mostly being fine or entangled metals. The non-ferrous metals (Al, Cu, Pb, etc.) can be recovered by eddy current separation, which also gives around a 50% recovery rate. The granulates are aged in open air to ensure immobilization of heavy metals and are then utilized as building materials, mainly as road subbase material [6,15].

According to Eurostat data, annual per capita generation of MSW in Belgium in 2015 was 475 kg (Figure 1), which translates to a total generation of 4.7 MT [16]. Thirty five percent of these wastes are incinerated in around 17 incineration installations [6]. In Flanders, 401 kT (Figure 2b) bottom ash are generated. A part of it is utilized in Flanders, a part outside Flanders and the rest outside Belgium; 174 kT bottom ash are processed, 24 kT as an alternative for gravel in Flanders; 72 kT are processed in Flanders and utilized in The Netherlands; 47 kT processed (in two treatment installations, one wet and one dry) and utilized in Germany; and 164 kT are landfilled [17]. Out of the processed fraction, 39 kT are used as alternative building sand in Flanders; 16 kT utilized in The Netherlands; and 40 kT are landfilled. To sum up, a total of 63 kT (15%) is utilized in Flanders; 134 Kt (34%) are utilized outside Flanders; and about 204 Kt (51%) are landfilled [18]. Utilization in Flanders is done as a road subbase material, landfill finishing material and elevation material for dike cores [17]. Furthermore, 134 kT are transported to the Netherlands and Germany due to stricter environmental regulations in Belgium, especially narrowed down by the allowable leaching levels of mainly copper (Cu) and at times zinc (Zn) and lead (Pb). The allowable content of Cu for shaped applications is 25 mg/m^2^ in Belgium, while the value is as high as 98 mg/m^2^ in Netherlands [19]. Possible beneficiation techniques to improve the utilization potential of these in specific applications are discussed further [13,18]. Treated bottom ashes also find application as aggregate for concrete blocks of dimension 150 × 75 × 40 cm that are lighter than the conventional concrete products [20]. It is to be noted that the bottom ash quantities described here are not only from municipal waste, but also from certain other non-hazardous waste streams. In Flanders, 62 kT of fly ash are used as an alternative building material. In Wallonia, part of the bottom ash that conforms to environmental stipulations is used in road construction, and the rest (usually exceeding the limit molybdenum content) is used in the cement industry [17].

## 3. Types of Incinerators and Ashes in Belgium

The quantity of wastes to be treated and the turbulence of waste or waste fuel mix undergoing incineration required determine the type of incinerator to be used. Types of incinerators used in MSW incineration plants are stationary hearth incinerators, rotary kilns, stationary and moving grates, modular/starved air systems, pyrolysis, gasification and fluidized bed systems [6]. A stationary hearth incinerator is used when the quantity of waste to be treated is very low, for instance in small-scale industrial, commercial and hospital incinerators. It has a refractory floor and air-inlet openings for complete combustion. Rotary kilns have been used in the past to burn MSW and are presently used to burn clinical wastes [6]. They consist of a steel cylinder lined with either refractory bricks or water tubes for cooling, inclined slightly towards the discharge end. Movement of the waste burned is controlled by the speed of rotation. The grate furnace is the most popular incinerator used now for mass burning of wastes. Types of grate systems include reciprocating grate, rocking grate, vibrating oscillating and impact grate, travelling grate and drum grate systems. Grates stay at a slope determined by the required residence time of the waste on the grates. The fluidized bed furnace consists of a homogeneous medium like sand, with air jets from below keeping the sand fluidized in high turbulence making the burn more homogeneous [21]. About 90% of the municipal solid wastes in Europe are incinerated in a grate incinerator [6]. All the incineration plants in Belgium have at least one grate furnace unit, and some have rotary kiln and a fluidized bed in addition to that [22]. There are 8 units with wet flue gas treatment systems, 9 with semidry and 2 with dry systems [6]. 

The composition of the residues depend on the input waste and the process parameters of the incinerator, even though it has little effect on the environmental quality of the material. The temperature profile in the incinerator has a large influence on the distribution of the volatile elements among different size fractions. Belevi and Langmeier found out that around 70% of zinc is transferred to the gaseous phase by 700 °C and about 80% by 800–900 °C. Eighty three percent of Sb is transferred to the gaseous phase at 500 °C and about 65% at 700 °C and 25% at 900 °C. About 67% Cd is volatilized at 700 °C, and about 86% is volatilized at 900 °C. The presence of chlorides enables the volatilization of heavy metals [23]. 

The ash or residue from the incinerator is named based on the point of collection of the ash from the incinerator. The International Ash Working Group (IAWG) recommended a nomenclature as given in Table 1. Large volumes of the total residues are composed of bottom ashes (more than 90%), and these contain least salts, heavy metals and POPs and are therefore best suited for recycling as construction material. Only 1–3% is fly ashes, and these are rich in chlorides, sulfates, PCDDs/PCDFs and other heavy metals, making them less suitable to be recycled [6]. Prior to recycling, all MSWI ashes require beneficiation treatments, the extent and intensity of which depend on the ash properties and the targeted use. 

## 4. Characteristics of Incineration Ashes

The physical and chemical characteristics of ash depend on the type and operating parameters of the incinerator in which it is produced. Hyks and Astrup observed changes in bulk composition of bottom ashes with the change of input composition, but neither a change in input composition nor operational parameters had an effect on the leaching of heavy metals from bottom ashes [27]. 

### 4.1. Physical Properties

Bottom ash has dry densities of 0.95–1.75 g/cm^3^ and a specific gravity of 1.1–2.7 [24,28,29,30,31,32,33]. The moisture content of the ash depends on post-combustion treatments and storage methods. Moisture is important for dust control and also for proper compaction. Loose bulk density of MSWI fly ashes decreases with decreasing ash separation temperature (temperature at which the fly ash is separated in the process flow) [34]. 

**Figure 3:**
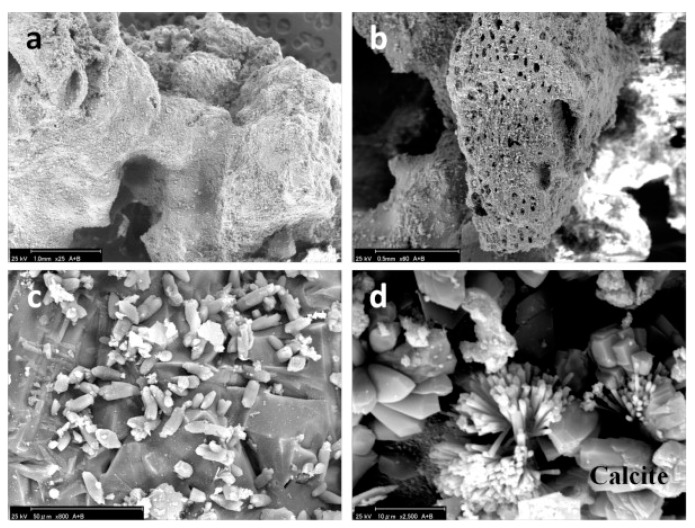
SEM images of bottom ash. (**a**,**b**) shows the irregularly-shaped bottom ash particles, which are porous in nature; (**c**) shows crystallized anhydrite or gypsum on the surface of bottom ash particles; (**d**) shows rhombohedrally-shaped calcite crystals and other calcium-based minerals [35].

**Figure 4:**
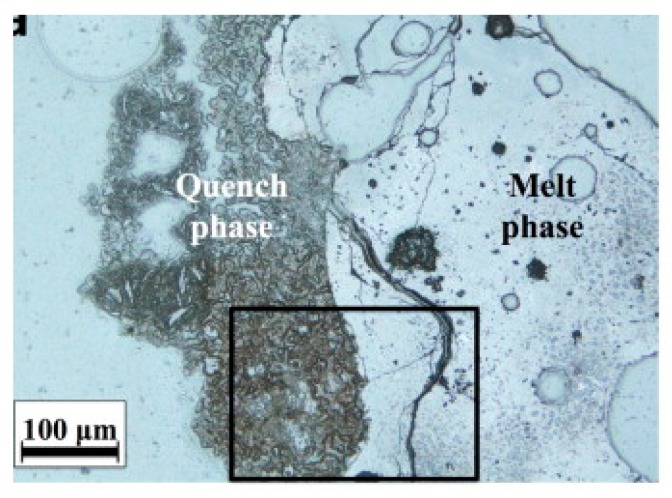
Photomicrographs of bottom ash particle differentiating the melt phase and quench phase [35].

The morphology depends mainly on the type of incinerator and the temperature profile to which it was subjected. Bottom ash is usually very heterogeneous and irregularly shaped and sometimes contains vesicles formed as a result of melting and quenching, as can be seen from the scanning electron microscopy (SEM) images in Figure 3. From photo micrograph analysis, bottom ash particles were distinguished into two classes: a melt phase composed of amorphous glass phases and a loose fragmented phase from water quenching, which is mainly composed of hydrates and carbonates, which can be seen in Figure 4 [35]. Inkaew et al. studied the effect of quenching of bottom ash on the mineralogy and morphology of bottom ashes. They point out that the main quench products are amorphous and microcrystalline calcium-silicate-hydrate phases, portlandite, hydrocalumite and Friedel’s salt, and they contain more chloride compared to other parts [36]. Image analysis indicates additional heterogeneities at the particle level with one particle often consisting of several phases with contrasting chemistries. Agglomeration, encrustation and rim formation add to the microstructural complexity and heterogeneity of the material [36]. In addition to phases formed during melting and quenching, bottom ashes show a large and heterogeneous residual fraction consisting of ceramic materials such as brick, stone, glass, ferrous and non-ferrous metals, non-combustible inorganic material and some residual organic matter [37]. For fly ashes, the morphology is affected by its separation temperature. The Blaine specific area of fly ash increases with decreasing separation temperature. At higher temperatures, the morphology of particles is more spherical. However, as temperature decreases, crystalline salts precipitate on silicate spheres, and irregular particles dominate [34,38].

### 4.2. Chemical Properties

#### 4.2.1. Chemical Analysis by XRF

**Figure 5:**
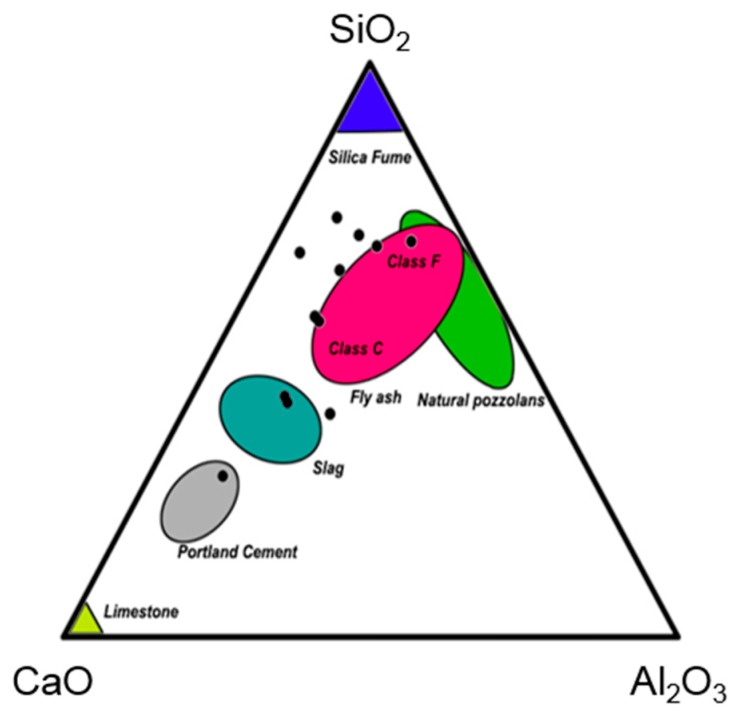
Composition of bottom ash from various incinerator facilities (black dots) superimposed on other materials commonly used in construction cf. [6,28,32,35,40,41,42,43,44,45,46,47,48,49,50].

Obviously, the bulk composition of residues varies from place to place and over time in an incinerator facility. The major chemical components in ash are CaO, SiO_2_ and Al_2_O_3_, which is illustrated in the Ca-Si-Al ternary diagram in Figure 5. In addition, minor components also have a significant effect on the technical and environmental quality of the material. The variation in minor components is due to the input waste characteristics and operational parameters [39]. Hyks and Astrup observed that the input of road salts and poly vinyl chloride (PVC) resulted in more chloride (Cl^−^), impregnated wood resulted in higher content of arsenic (As), automotive shredder residues induced more barium (Ba), copper (Cu), molybdenum (Mo), nickel (Ni), antimony (Sb), tin (Sn) and zinc (Zn) and batteries increased the content of cadmium (Cd), cobalt (Co), sulfur (S) and strontium (Sr) [27]. The increase in combustion temperature results in the increase of pH due to conversion of calcite to portlandite [17]. The fly ash depicted in Figure 5 is that from thermal power plants and not from incinerators. This shows the bottom ash compositions superimposed on compositional regions of other residues that are commonly used as construction materials. Clearly, the bottom ashes cover a wide range of compositions that are closest to that of iron blast furnace slags and of coal combustion fly ashes.

The chemical composition of MSWI fly ashes depends on their separation temperature from the flue gas line. Keppert et al. studied the effect of separation temperatures on the chemical composition of three fly ashes with separation temperatures higher than 700 °C, between 500 and 700 °C and between 250 and 300 °C. It was observed that the content of soluble salts (Cl^−^ and SO_3_^2−^) and most heavy metals (As, Cd, Pb, Sb) increases with decreasing separation temperature [34].

#### 4.2.2. Mineralogical Composition

The mineralogical composition often determines the technical properties and the end-use of a resource. Relative proportions of the constituent minerals are the underlying factors explaining why some materials are suitable for use as a reactive cement component and others are not. The major and minor mineral constituents reportedly found in bottom ashes, fly ashes and sintered ashes and their significance in various utilization scenarios are tabulated in Table 2. Ageing effects major changes in mineralogy, including the formation and increase of minerals such as calcite, quartz, sulfates and ettringite [48]. Ettringite and hydrocalumite are formed due to the quenching of bottom ash. 

Apatite group minerals are formed in ashes as a result of phosphation. They are stable minerals, and during their formation, they encapsulate heavy metals. Gypsum and anhydrite are not inert; for instance, when used along with cement, they will affect the hydration reaction unlike most of the other crystalline minerals present in ash.

## 5. Beneficiation Needs for Use of MSWI Ash in Building Materials

### 5.1. Metallic Aluminum and Zinc

The various sources of aluminum in municipal waste include beverage cans, aluminum foils, nails, etc., and as a result of attrition within the waste, their size gets reduced greatly. The eddy current separation technique (cf. infra) can be used to recover the aluminum particles from the waste in incineration plants. However, small particles will not be recovered and will thus remain in the ash. When it is mixed in together with alkaline water, e.g., for utilization as aggregate in concrete or as a supplementary cementitious material, the aluminum in ash will oxidize and release hydrogen gas. This gas formation results in a high porosity of the matrix, and if continuous moisture is supplied, the reaction will continue after concrete hardening and result in spalling and cracking. The resulting aluminum hydroxide generated was reported to exist in different forms in the matrix: amorphous Al(OH)_3_, bayerite, gibbsite and boehmite [46]. It is reported that the structure of the product formed depends on the rate of crystallization. Rapid crystallization leads to the formation of bayerite, and slow crystallization leads to the formation of gibbsite [53]. Elemental Zn present in ash also reacts similarly to generate hydrogen gas and the different stoichiometrical equations of the reactions are given below [41,54,55,56]

(1) $$\mathrm{Al}+2{\mathrm{OH}}^{-}+H_{2}O\;\to {\left[\mathrm{AlO}{\left(\mathrm{OH}\right)}_{2}\right]}^{-}+H_{2}↑\;\mathrm{pH}>7$$

(2) $$2\mathrm{Al}+\;2{\mathrm{OH}}^{-}+6H_{2}O\;\to 2{{\left[\mathrm{Al}{\left(\mathrm{OH}\right)}_{4}\right]}^{-}}_{\mathrm{aq}}+3H_{2}$$

(3) $$\mathrm{Zn}+2{\mathrm{OH}}^{-}\;\to {{\mathrm{ZnO}}_{2}}^{2-}+H_{2}$$

(4) $$2\mathrm{Al}+\mathrm{Ca}{\left(\mathrm{OH}\right)}_{2}+2H_{2}O\;\to \;\mathrm{Ca}{\left({\mathrm{AlO}}_{2}\right)}_{2}+3H_{2}$$

CUR (Civieltechnisch Centrum Uitvoering Research en Regelgeving) recommendation 116:2012 for use of MSWI bottom ash as aggregate in concrete regulates the content of elemental aluminum in the ash to a maximum of 1% by mass of bottom ash. It recommends a test set up to quantify the equivalent elemental aluminum content by measuring the volume of hydrogen released on reaction with 1 M sodium hydroxide solution [56]. It is called an equivalent aluminum because zinc even though present in smaller quantities undergoes a similar reaction, and the hydrogen collected is generated by aluminum and zinc jointly. However, it can be seen from Table 3, which is a theoretical calculation conducted by the authors, that the volume of hydrogen produced is significant even at a concentration of 0.1%. A certain part of this gets trapped inside concrete and reduces the strength by creating porosity.

### 5.2. Salts

Salts are mainly present in the form of chlorides and sulfates and, in combination with heavy metals, render MSWI fly ash hazardous, which prevents its utilization. It is rarely present in the bottom ash fraction, especially the coarser ones. The chloride content varies between 0.5% and 15% in fly ash and 0.2% and 5% in bottom ash. Most of the chlorides in the bottom ash are present in the finer size fractions [57]. This is due to metal chlorides and sulfides having a lower boiling point than their oxides and phosphates and thus becoming volatilized in the incinerator [58]. These mostly exist as soluble salts and will cause leaching problems when landfilled. Crystalline chlorides in ash exist as halite, Friedel’s salt and hydrocalumite [35]. When ash is used as a supplementary cementitious material, chloride ions released into pore solution accelerate the corrosion of steel and comprise a great threat to steel-reinforced concrete durability [59]. Free chlorides present in concrete react with Ca(OH)_2_ to form calcium chloride and magnesium hydroxide, which eventually leaches out, exposes reinforcements and also leads to their corrosion [44]. Furthermore, when the chloride content in pore solution exceeds a critical value, the passive layer on the steel reinforcement disintegrates, and localized pitting corrosion is caused.

(5) $$2{\mathrm{Cl}}^{-}+\mathrm{Ca}{\left(\mathrm{OH}\right)}_{2}\;\to {\mathrm{CaCl}}_{2}\left(\mathrm{leakage}\right)+2{\mathrm{OH}}^{-}$$

When high amounts of sulfates are present, the reaction of aluminate and calcium sulfates forms ettringite and monosulfate. Washing with water is the easiest solution to remove chlorides and sulfates, but it does not remove bound chlorides. To remove chlorides further, thermal treatments such as sintering, roasting and calcination have been tested. A single technique was not sufficient for maximum removal. A combination of thermal treatment and washing was able to remove about 90% of chlorides [35].

### 5.3. Heavy Metals

The presence of heavy metals in MSWI ash and its leaching into ground water have been a problem for a long time, since it was landfilled or used as a subbase material in road construction. Many countries are adopting source reduction techniques by limiting the toxic trace metals in different products. The heavy metals usually present in MSWI ash are arsenic (As), barium (Ba), cadmium (Cd), chromium (Cr), copper (Cu), molybdenum (Mo), nickel (Ni), lead (Pb), tin (Sn), antimony (Sb), selenium (Se) and zinc (Zn). The leaching of heavy metals is less affected by the change in bulk composition of ash or operating parameters of the plant [27]. Heavy metals are more cumulated in fly ashes than bottom ashes, except Cu, Cr and Pb due to their low volatilities [33,38]. In Flanders, the leaching of various heavy metals is regulated by a column leaching test done according to CMA/II/A.9.1 based on European Committee for Standardisation/Technical Specification, CEN/TS 14405:2004. Both procedures are different mainly in the maximum particle size (4 mm for CMA/II/A9.1 and 10 mm for CEN/TS 14405:2004) and column diameter (5 cm for CMA and 10 cm for CEN). The utilization of bottom ashes in Flanders is limited by the cumulative release of different species at a liquid to solid ratio of 10, and the limits are specified in VLAREMA (Vlaams reglement betreffende het duurzaam beheer van materiaalkringlopen en afvalstoffen). The round robin test reported by Geurts et al. verified the reproducibility of the test to confirm whether it complies with legal limits, even though the reproducibility of absolute leaching values of Cu and Zn was questionable. To achieve greater reproducibility, they recommend using an identical analysis technique for the eluates [60]. Utilization of bottom ash in Flanders is mainly limited by its Cu, Zn and Pb contents being past allowable limits as can be seen from Table 4. Some of these materials are transported to The Netherlands or Germany where more tolerance for these metals exists [61]. 

The presence of metallic aluminum was found to have an effect on the leaching of chromium. The presence of Al in the (0) oxidation state appeared to prevent leaching that happens by reduction of Cr(VI) to Cr III, but the presence of manganese oxides accelerates leaching. This is a conflict of interest since the presence of Al is detrimental to the strength of concrete while advantageous in the case of leaching [64]. 

### 5.4. Persistent Organic Pollutants

Certain fly ashes from MSWI plants are classified as hazardous waste due to their dioxin and other POP content. Polychlorinated dibenzo-p-dioxins (PCDDs) and polychlorinated dibenzofurans (PCDFs) dioxins are polychlorinated biphenyls present in MSWI ashes. The most poisonous one is 2,3,7,8—tetra chloro benzo dioxin (TCDD) [65]. They can be either present in the feed, formed from chlorinated precursors such as PCBs, chlorophenols, chlorobenzene, etc., or formed spontaneously from the organics present in the fly ash, by chlorination and oxidation [66]. These are highly toxic substances. If inhaled, they are carcinogenic. Human exposure to these hazardous contaminants is possible by either inhalation of dust or ingestion of contaminated ground water [67]. Even though these compounds are formed by chlorination, the chlorine content in the input has little effect on the quantity of PCDD/Fs compared to the design and operating parameters of the plant [68]. As volatile compounds, they are removed from stack gas using activated carbon as the adsorbent and bag house filtration [69,70]. The addition of chemical suppressants after combustion is an effective method to reduce their formation. Various sulfur and nitrogen compounds have been identified as effective suppressants. In accordance with the Basel Convention on the Control of Transboundary Movements of Hazardous Wastes and Their Disposal of 1992, the limit for atmospheric emission of PCDDs is 0.14 ng total toxic equivalent (TEQ)/Nm^3^ at a reference oxygen content of 11%. Total toxic equivalent (TEQ) corresponds to the sum of different cogeners multiplied by their toxic equivalency factor (TEF) values [65].

According to the same source, the limits for waste to be classified as low POP content are:

- PCBs: 50 mg/kg
- PCDDs and PCDFs: 15 µg TEQ/kg
- Aldrin, chlordane, DDT, dieldrin, endrin, heptachlor, hexachlorobenzene (HCB), mirex and toxaphene: 50 mg/kg for each of these POPs.

### 5.5. Amorphous Silica Content

The amorphous silica content is an obstacle only when incineration ashes are used as aggregate in concrete. The glassy components in ash and alkali in cement react to form alkali silica gel, which has a very high volume and thus causes cracking. However, when metallic aluminum is present, it forms voids in the cement matrix as explained above; the gel formed as a result of alkali-silica reaction occupied these voids, and no cracks were generated, as reported [71].

## 6. Bottom Ash Pre-Treatment Techniques

MSWI ash is not an industrial product, but a by-product of the incineration of municipal solid wastes. Therefore, it is difficult to control its properties during production, and beneficiation of ash is mandatory for recycling. Metal recovery is the most important treatment step, as it is advantageous both for the economy of the plant and for enabling further use of the ash as a cementitious material. Berkhout et al. reviewed various techniques for metal recovery [72]. Other reasons for pre-treatment are the obstacles specified in Section 5. Some of the pre-treatment techniques are currently employed at an industrial scale, and others are still in the research stage. 

### 6.1. Pre-Treatment Techniques Used at the Industrial Scale

#### 6.1.1. Magnetic Separation

This is the most popular technique to separate materials based on their magnetic properties, ferromagnetic, paramagnetic or diamagnetic, and also their degree of magnetism. There are different types of magnetic separation techniques involving cross-belt magnetic separators, drum magnetic separators or magnetic pulley separators [73]. The magnetic density separation (MDS) technique can be used to separate the aluminum from other non-ferrous heavy metals based on their difference in apparent density in a gradient magnetic field [72]. It is presently used to separate larger fractions, but not employed in the treatment of other finer residues from the incinerator. It is reported that the efficiency of ferrous metal recovery by magnetic separation is as high as 57–83% [74]. De Boom et al. demonstrated the application of this technique to MSWI boiler fly ashes to recover magnetic particles enriched with Cr, Fe, Mn and Ni [75].

#### 6.1.2. Eddy Current Separation

Eddy current separation (ECS) is the preferred method used in incineration plants and waste sorting facilities to separate the non-ferrous metals like aluminum from waste and ash. It has limited efficiency when applied to wet ash. Its efficiency depends on the size of the particles and ranges from up to 100% efficiency for particle sizes greater than 20 mm, down to 0% for those less than 5 mm. The efficiency can be improved by increasing the number of screening steps. On average, the efficiency of aluminum removal from MSWI ash by ECS is around 30% [46,74,76].

#### 6.1.3. Washing

Washing is the simplest beneficiation technique to remove many of the deleterious compounds in MSWI ash preventing its utilization. Washing in alkaline conditions hydrates elemental aluminum and removes certain heavy metals and in acidic conditions removes certain other heavy metals and chlorides and sulfates. Since the washing process carries with itself the huge baggage of secondary pollution of water, some waste water streams have also been tested for this purpose, which resulted in some additional advantages including binding of heavy metals, which are discussed in further sections. 

The presence of soluble leachate salts cuts the utilization potential of MSWI ashes. Water washing techniques have been tested for their effectiveness to remove excess chlorides and sulfates and also heavy metals present especially in the fine fractions. Many have reported that the chloride content in ashes can only be reduced to 0.5% by washing due to the presence of insoluble chlorides [35,77,78,79]. Chen et al. studied the effect of washing with water saturated with calcium hydroxide to control the ionic equilibrium of calcium ions and thus facilitate the dissolution of all chlorides except calcium chloride going into solution [78]. This prevents calcium compounds getting washed away, which would be a disadvantage when used as raw material for cement production. High waste water generation is the main disadvantage of this technique. 

#### 6.1.4. Shaking Table

Shaking tables/wet tables such as Wilfley tables are density separation technologies that separate heavy metal particles from other lightweight particles [80]. They have a sloping plank with ribs on the surface. Water/slurry flows perpendicular to the ribs, and the table oscillates parallel to the ribs. The technique is used at the industrial scale, and it successfully separates precious metal particles such as tin, copper, gold, lead, zinc, tungsten, etc., of a size of 50 µm–2 mm [81,82].

#### 6.1.5. Jig Head Separation

These are devices to separate materials in wet condition that works on the principle for density separation. The particles are pushed upwards by pulsating motion, and when the pulse in water drops, the particles settle in order of their densities and thus can be separated. A tank filled with the slurry to be separated is pulsated up and down, and this results in settling of denser particles of gold, chromium, lead, etc., of a size of 75 µm–6 mm, which are collected and separated [81,82].

#### 6.1.6. Ageing

Ageing is an effective and widely-used technique to reduce the leaching of heavy metals. It is a combined process of hydration, carbonation and oxidation. It is an exothermic process and raises the temperature of bottom ash from 70–80 °C [17]. During ageing, more minerals such as ettringite, hydrocalumite, C-S-H, carbonates, sulfates, etc., are formed, which bind heavy metals, thus reducing leaching. Sulfate minerals have lesser stability compared to carbonates and hydroxides [48,49].

### 6.2. Pre-Treatment Techniques in the Research Stage

#### 6.2.1. Washing with Alkali

Higher alkalinity accelerates the oxidation of aluminum to form hydrogen and also increases the solubility of sulfates [83]. The disadvantages of alkaline washing include the dissolution of fine reactive particles, leading to some loss of cementitious properties during the washing process, and also the high cost of alkali.

#### 6.2.2. Sulfide Rich Effluent

Effluent from waste water treatment contains sulfides generated by sulfate-reducing bacteria. When ash is treated with this sulfide-containing water, the heavy metals are precipitated as insoluble sulfides and are thus stabilized. The stabilization effect of anaerobic effluent also acts through carbonation, by CO_2_ dissolved in the effluent. It is reported that the treatment can stabilize Ca, Cu, Pb, S and Zn, whereas it increased leaching of P and had no effect on As, Cr and Mo [84].

#### 6.2.3. Wet Grinding 

For utilization of ash as supplementary cementitious material, grinding of bottom ash is required to break it down to particle sizes comparable to that of cement. If the grinding process is done in wet condition, it provides enough water, turbulence and dissolution of alkaline phases like Ca(OH)_2_, which gives enough pH for the aluminum reaction to occur. Bertolini et al. have reported that the 180-day compressive strength of concrete increased by four-fold compared to that of dry ground ash and was 25% higher than that of 100% ordinary Portland cement (OPC) mix. This was mainly because of the removal of expansive Al [7].

Further, wet milling is identified as an effective technique to stabilize certain heavy metals such as Pb. Wang et al. reported the effect of wet milling in stabilization of Cr, Cu, Zn and Pb for ash from grate furnaces and of Cr and Zn for ash from fluidized bed incinerators without a water extraction procedure [85]. Chen et al. reported stabilization of heavy metals except Cr by wet grinding in a liquid to solid ratio of nine at 93 rpm for 96 h. Wet grinding reduced the median diameter of particles (d50) of ashes from 72.5 µm down to 3.95 µm, as well as the crystallinity of the material. Concurrent fragmentation and agglomeration of the particles results in stabilization of heavy metals [86].

#### 6.2.4. Carbonation

Carbonation is an effective technique to immobilize the heavy metals, if the ash needs to be disposed safely, or when used as an aggregate when it is not milled. Arickx et al. studied the effect of carbonation and different parameters related to it on leaching of heavy metals. Stack gas from the incinerator was also used as a source of CO_2_. However, for applications that involve milling, carbonation does not prove to be very effective in immobilizing heavy metals [87]. Aguiar del Toro et al. report an increase in mobility of chloride ions as a result of carbonation [88]. Chlorides exist in MSWI ash, both as soluble salts such as NaCl, KCl, etc., and also in insoluble forms, such as Friedel’s salt (3CaO·Al_2_O_3_·CaCl_2_·10H_2_O) and calcium chlorocalumite (11CaO·7Al_2_O_3_·CaCl_2_) in heated ashes [35]. Chlorides existing as insoluble Ca-salts can be disintegrated by carbonation and then can be removed by washing.

#### 6.2.5. Phosphation

Phosphation is the process of converting heavy metals into their insoluble phosphate form to prevent leaching into ground water. As a result of phosphation treatment, apatite group minerals are formed. They encapsulate the heavy metals present in the ash and thus stabilize them. Carbon dioxide gas evolves during phosphate reaction. The reaction also results in a reduction of pH, even though fly ash with high calcium hydroxide content is reported to maintain high pH [89,90].

#### 6.2.6. Cement/Other Binder Stabilization

Cement-based stabilization is a widely-used technique to prevent toxic element leaching in landfills. It converts the liquid or loose waste into a monolithic form and also stabilizes chemically-reactive species into more stable forms. The high surface area of C-S-H also facilitates adsorption of species preventing leaching. It has been reported that Cr ion stabilization is most effective in binders with slag replacement. The stabilization is materialized by substitution in calcium aluminate hydrates. Ettringite stabilizes heavy metal oxyanions like chromate, arsenate and selenite. Even though cement stabilization is a technique presently used for disposal of ashes, the binding effect indicates the potential for reuse without the harmful effects of heavy metals [91,92,93]. Galiano et al. used coal fly ash-based geopolymers to stabilize fly ash and compared with other classical stabilizers like cement, lime, slag and metakaolin. Zn, Sb and Sn showed effective immobilization, whereas Mo, V and Cr were not immobilized much. The best immobilization was demonstrated by OPC, lime and a mix of blast furnace slag and potassium silicate [94]. Bosio et al. employed a mix of colloidal silica or rice husk ash with coal fly ash for stabilization of MSWI fly ash. Both showed considerable and comparable stabilization of heavy metals [95,96].

#### 6.2.7. Hydrothermal Treatment

Hydrothermal treatment is the process of solidification by application of steam under high pressure. It results in the formation of new stable minerals such as tobermorite, katoite or C-S-H, and the composition should be optimized (Ca/Si ratio of 0.83) for maximum generation of tobermorite and thus maximum strength. Jing et al. reported a positive effect of the addition of NaOH in hydrothermal solvent on the strength [97]. The same researchers investigated the result of hydrothermal solidification of MSWI bottom ash mixed with quartz, slaked lime and water cooled blast furnace slag [98]. The hydrothermal solidification of MSWI bottom ash without any additives was studied, since it had an optimum Ca/Si ratio inherently [99]. Furthermore, they studied the effect of hydrothermal treatment in solvent with a mixture of ferric and ferrous sulfate on dioxin decomposition [100]. High temperature and ferric and ferrous sulfate promote decomposition, temperature having the highest effect. Bayuseno et al. studied the effect of hydrothermal solidification of MSWI fly ashes on heavy metal fixation. They washed the fly ash, subsequently mixed with alkaline solution and treated in an autoclave at 90–180 °C. This resulted in the formation of minerals such as tobermorite and katoite and a marked reduction in leaching of heavy metals [101].

An optimized wet extraction of heavy metals and chlorides in hydrothermal conditions was experimentally tested by [88] with acid extraction and carbonation. The liquid to solid ratio, carbonation and leaching time for maximum extraction were optimized. It was observed that hydrochloric acid was most effective in the extraction of metals [102].

#### 6.2.8. Thermal Treatment

Calcination of the ashes at a temperature above 600–800 °C eliminates the presence of toxic organic compounds like poly-chlorinated dibenzo-p-dioxins (PCDDs) and polychlorinated dibenzo furan (PCDFs). Furthermore, calcination may result in oxidation of Cr(III) to Cr(VI), which is the soluble form of chromium and makes it more prone to leaching. Therefore, application of a calcination process should be done with enough care to avoid leaching of Cr(VI) [25]. Furthermore, heating above 600–800 °C disintegrates the quench phase and results in volatilization of chlorides [35]. Reaching a maximum temperature of above 900°C is necessary to avoid reformation of PCDD/Fs. 

Washing of ground ash at comparatively high temperatures (65–80 °C) can accelerate the process of metallic aluminum hydration, thus mitigating its harmful effects if happening during utilization [103].

#### 6.2.9. Electrodialytic Remediation

Electrodialytic remediation is a technique that combines the technique of electro-dialysis with the electro-migration of ions, explored for removal of heavy metals from different kinds of waste streams and affected materials. Pedersen studied the effect of different assisting agents (deionized water, 2.5% NH_3_ solution, 0.25 M citric acid and 0.25 M ammonium citrate) in the extraction efficiency of five heavy metals (Cd, Pb, Zn, Cu and Cr) from MSWI fly ash. The optimized removal of all five heavy metals was obtained when ammonium nitrate was used as the assisting agent [104].

#### 6.2.10. Revasol Process

This is an optimized process developed by Solvay Company (Neder-Over-Heembeek, Brussels, Belgium) in collaboration with Université Libre de Bruxelles (ULB) (Brussels, Belgium) in Belgium to increase the utilization of MSWI fly ash. The process consists of three steps [52]: water dissolution, in which the salts are dissolved in water without allowing heavy metals to get dissolved; phosphation with phosphoric acid, which stabilizes heavy metals; and calcination to remove organic impurities present. 

Aubert et al. modified the process with the addition of Na_2_CO_3_ in the washing step to accelerate the oxidation process of aluminum and also to increase the solubility of sulfates [26].

Table 5 summarizes various obstacles and methods already employed and under research to resolve them.

## 7. Research Regarding Ash Pre-Treatment in Belgium

Various research projects are carried out within the Strategic Initiative Materials under the MaRes program (Materials from solid and liquid industrial process Residues) that aims at creating a toolbox to separate useful fractions from waste streams and valorizing them in building materials. The ASHCEM project deals with developing novel cements and building materials from municipal solid waste incineration ashes. The SUPERMEX (sustainable transformation of new and landfilled solid industrial residues into metals and inorganic polymers using a SUPERMetalEXtractor) project investigates sustainable transformation of new and landfilled solid industrial residues using a SUPERMetalEXtractor; while the SMART (Sustainable Metal Extraction from Tailings) project generates a toolbox for the extraction of metals from various tailings [105].

Various other research groups in Belgium also investigated various pre-treatment techniques to improve the utilization potential of the residues. Van Gerven et al. studied the effect of carbonation in reducing heavy metal leaching [87,106]. Saikia et al. studied the effects of various treatments including heat treatment and treatment with sodium carbonate to curb the harmful effects of elemental aluminum and sulfates [41]. Arickx et al. studied the effect of accelerated carbonation on curbing leaching of heavy metals and identified that around four weeks of accelerated carbonation leads to the Cu leaching being under the limits. They also conducted accelerated carbonation using the stack gas from the incinerator, and condensate formation due to high humidity of stack gas was identified as the major hindrance towards its application [87]. The Revasol process was developed to render MSWI ashes non-hazardous and fit to be utilized by reducing the soluble content, reducing the leachable content of heavy metals and destroying POPs [52]. Van Den Heede et al. studied the effect of washing with NaOH and found it to be effective to reduce the metallic aluminum reaction when used as an aggregate to manufacture Lego bricks [71].

## 8. Utilization of MSWI Bottom Ash in Building Materials

**Figure 6:**
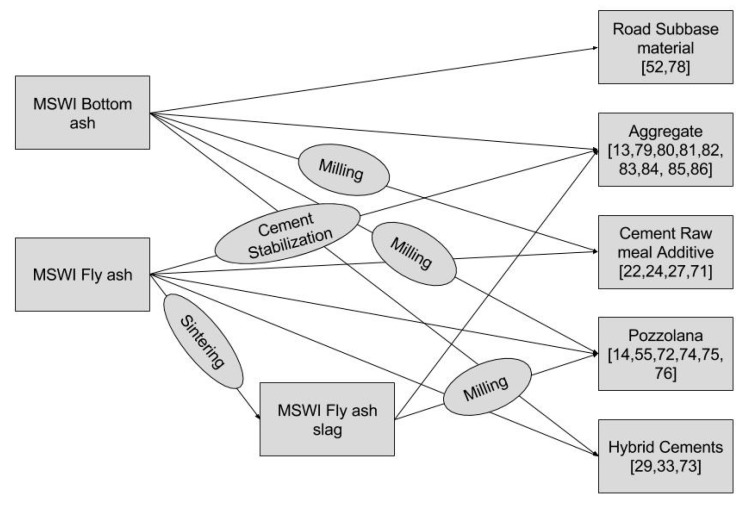
Routes of valorization for MSWI ash.

Bottom ash has potential to be used in various applications, after specific treatments for each application, as depicted in Figure 6. It is presently used for certain applications such as in road construction, cement production as additive, concrete production as aggregate etc., which is discussed in detail further.

### 8.1. Present Areas of Utilization

At present, MSWI ash is mainly utilized as a road subbase material, landfill structure material, embankment fill, as cement raw material and concrete products, out of which, the use in road base applications tops the lot in terms of volumes [19]. 

#### 8.1.1. As a Road Construction Material and Landfill Stabilizer

Bottom ash can be used as aggregate in foundation layers, sub-base, embankment and as capping material in road construction [107]. When it is used in the hydraulically-bound form, washing of ash is done prior to utilization to prevent heavy metal leaching at the point of utilization. Since this does not involve milling of ash, the washing process can be more effective in curbing aluminum-induced expansion [77,108]. When used in bitumen bound applications, it resulted in higher bitumen demands due to the porosity of the material compared to conventional aggregates [107].

In landfills, it is used as a stabilizing layer to protect the geomembrane, intermediate layer and also as the leachate drainage layer [109,110].

#### 8.1.2. As Cement Raw Meal Additive

The constituents of cement raw meal are calcium oxide, silica, alumina and iron oxide as the main components, along with other minor constituents, with specific limits to each of these. An appropriate composition is achieved by carefully proportioning different natural mineral sources like limestone, clay, etc. Presently, different alternate raw materials are added to the raw meal, usually as sources of silica. The replacement level is limited because of the low Ca content or the presence of other deleterious materials like alkalis and chlorine. Since cement is produced in huge quantities, utilization in even limited fractions can result in huge consumption of the MSWI ashes. A part of heavy metals, Cu, Zn and Pb, which is mostly affecting the utilization of bottom ashes, can be safely incorporated in mineral phases in clinker without affecting its properties. The proposed safe limits of Cu and Zn in clinker are 0.35% and 0.7%, respectively [111].

Eco-cement is a cement developed and being produced in bulk quantities in Japan with incineration ash mixed with limestone and other additives to adjust its composition being used as raw material for Portland cement production. Excess heavy metals in the ash are vaporized as chlorides and are collected in baghouses, and the heavy metals are regained from these by smelting, facilitating maximum recycling efficiency. Two types of eco-cements are being produced, normal Portland cement with alite, belite, aluminate, ferrite and calcium sulfate and a rapid hardening type with alite, belite, ferrite, C_11_A_7_ and CaCl_2_ [112].

Replacement of cement raw meal by incineration bottom ash up to 15% was investigated by Shih et al. [42]. Five raw mixes were investigated: classic raw meal, raw meal with 3%, 10%, 15% replacement by bottom ash and with 15% magnetically-repelled MSWI ash after magnetic separation treatment. One mix was conditioned to required oxide proportions by the addition of pure calcium oxide. The lime saturation factor (LSF) of mixes were 1, 0.95, 0.84, 0.77 and 1.01 for 0%, 3%, 10%, 15% replacement and conditioned mix, respectively. Various literature works specify the required range of LSF as 0.92–1 [113]. A reduction in cement strength was observed when the lime saturation factor (LSF) was lower due to a lack of calcium for the formation of tri-calcium silicate. Higher strength was observed when additional calcium oxide was added to the system. A similar study with replacement of 5% and 10% of raw meal by dried and milled bottom ash without adjustment for the LSF was conducted by Krammart and Tangtermsirikul [32]. This replacement resulted in increased belite content at the expense of alite, resulting in a decrease in compressive strength and a delay in setting. Kikuchi produced Portland cement from raw meal with MSWI ash, sewage sludge, sewage dry powder, aluminum dross, limestone, clay and copper slag with up to 40% of MSWI fly ash. Significant fixing of chlorine in the form of calcium chloroaluminate was reported. Furthermore, the phosphate content in MSWI ash reduced the burnability of the mix and resulted in lower conversion of belite to alite [44]. Wang et al. manufactured clinker using washed fly ash as a component up to 15% with LSF, SM (silica modulus) and AM (alumina modulus) adjusted to prescribed limits. Washing of fly ash was optimized for maximum removal of chlorides and sulfates. Cement phases produced by sintering and the compressive strength of mortars were comparable to that of conventional raw meal [114].

#### 8.1.3. MSWI Bottom Ash as Aggregate in Concrete

Research regarding use of bottom ash as aggregate in concrete started around two decades ago [55]. CUR Recommendation 116:2012 recommends utilization of MSWI bottom ash with less than 1% aluminum content to be used in concrete [56]. The main difficulties faced were expansion due to elemental Al, high water absorption, which increases water demand of concrete, and alkali-silica reaction. Washing with alkali, such as sodium hydroxide, is a possible solution for reducing expansion due to metallic aluminum. 

Despite the mentioned practical challenges regarding the use of bottom ash as aggregate in concrete, research has been done on how a full replacement of traditional concrete aggregates by bottom ash-based aggregates would be attainable for certain concrete products, also in Belgium. For instance, Van den Heede et al. investigated whether non-steel reinforced prefabricated concrete Lego bricks of an acceptable quality could be produced with treated bottom ashes originating from a Belgian MSWI company as full aggregate replacement for limestone 2/6 and 6/20 [83]. It was found that the reduced concrete workability due to the high water absorption of the bottom ash can be dealt with by subjecting the fine bottom ash fraction to a crushing operation to eliminate the porous elements and by pre-wetting the fine and coarse bottom ash fractions in a controlled manner prior to concrete mixing. To overcome unacceptable expansion and longitudinal void formation related to the elemental aluminum present in the bottom ash, a reactive washing step with 1 M NaOH was found to be very effective. A modified Oberholster test indicated that the applicable 0.1% expansion limit was not surpassed. The required concrete strength class (C20/25) for prefabricated Lego brick production could be achieved without any problem [71]. Collivignarelli and Sorlini investigated the use of cement-lime stabilized MSWI fly ash as recycled aggregate in concrete. They reported to have achieved more than minimum compressive strength and good environmental compatibility with the tested mixes [115]. Results from the manufacture of lightweight aggregates with MSWI ash and reservoir sediment as additives are reported. The dosage of MSWI ash was 30%. The density of the aggregates manufactured was 0.99 g/cm^3^ [116]. Ferraris et al. studied the effect of utilization of bottom ashes vitrified at 1450 °C without any agents as filler, sand and aggregate in concrete. Slump, alkali silica reactivity and mechanical properties were evaluated. When used as a filler, the 150-day compressive strength remained the same as that of a reference up to 20% replacement of cement and up to 75% effective replacement of natural aggregate [117]. Cioffi et al. studied the properties of artificial aggregates manufactured by binder stabilization of bottom ashes. These bottom ashes did not conform to the Italian environmental limitations for utilization. However, after stabilization, the cumulative leaching value of artificial aggregates conformed to environmental limitations. Moreover, the concrete blocks made with these aggregates conformed to the requirements for lightweight structural concrete [118]. Hwang et al. manufactured lightweight aggregates from reservoir sediment with MSWI fly ash as an additive. Concrete manufactured from these lightweight aggregates was tested for various properties such as compressive strength, electrical resistivity and ultrasonic pulse velocity, and it was concluded that the maximum addition of fly ash should be limited to 30% [119].

### 8.2. Potential Areas of Utilization

In addition to the low value applications except for as raw meal additive, for which bottom ashes are presently used, there are ongoing and past research works studying its application in higher value applications, which requires more pre-treatments to be done.

#### 8.2.1. MSWI Ash as a Pozzolanic Addition in Cement

Supplementary cementitious materials are inorganic materials that, when used in conjunction with Portland cement, contribute to the properties of the hardened concrete through chemical reaction, e.g., hydraulic or pozzolanic activity. SCMs are mainly classified based on their origin as natural, thermally-activated and by-product SCM. The most used by-product SCMs include blast-furnace slag, fly ash from coal combustion and silica fume. These SCMs differ from each other in relative percentage of constituents, mineralogy, kinetics of reaction and also in the hydration products formed. ASTM 989 specifies the required fineness for use of slag as a pozzolana in terms of maximum percentage retained when wet screened on a 45-µm sieve to be a maximum of 20%. NBN 450-1 (Bureau voor Normalisatie, Belgium) sets the value to a maximum of 40% and 12% for two categories. Like slags, bottom ashes are generated as granulates and need a grinding step prior to their utilization as a supplementary cementitious material to reach this fineness. Like Portland cement, the main constituents of supplementary cementitious materials are CaO, SiO_2_, Al_2_O_3_ and Fe_2_O_3_; however, most SCMs are enriched in silica compared to PC. NBN EN 450-1 and ASTM C 618 specify a minimum total content of 65% and 70% respectively for silica, alumina and iron oxide [120]. NBN EN 450-1 also limits the total organic carbon content to 5%, 7% and 9% for Category A, B and C fly ashes, respectively. For bottom ashes in Belgium, it is limited to 3% for ensuring burnout and is usually present in the range of 0.5–2.4% [17]. This limits free lime, alkali, chloride, sulfate and phosphate contents to 1.6%, 5.5% Na_2_O_eq_ 0.1%, 3.5% and 5.5% by mass, respectively [121]. The washing and ageing in moist condition steps, which are already done in the field, converts the free lime present now into calcium hydroxide and eventually calcite. ASTM C 150 limits the maximum content of alkalis versus total binder to 0.6% expressed as sodium oxide equivalent (Na_2_O_eq_) when used in conjunction with reactive aggregates [122]. ASTM 989 limits the content of sulfur present as sulfide to a maximum of 2.5% and sulfate to a maximum of 4.0% in slag used in concrete [123]. MSWI ash is also a potential addition to the list of SCMs once the potential issues are resolved, which includes the presence of metallic aluminum, zinc, salts and heavy metals.

Remond et al. studied the effect of the replacement of cement by MSWI fly ashes on setting times, compressive strength, flow and microstructure of mortar and concrete. The pozzolanic phases were identified as CAS_2_ (calcium aluminodisilicate) and AS (alumina silicate). The presence of zinc and lead resulted in the increase of setting times, and the presence of chloride had an accelerating effect, which increased the seven- and 28-day strengths for up to 15% ash replaced samples [124].

An optimized treatment procedure called the Revasol process and a modified process to eliminate metallic aluminum and sulfate were employed to get two treated fly ashes, which were further tested for reactivity and hydration as a pozzolanic addition by Aubert et al. They modified the previously developed Revasol process by including washing with Na_2_CO_3_ solution. Calcium aluminate hydrate, ettringite and calcium carbo-aluminate were generated as hydration products on reaction with lime. The modified treatment processes employed were successful in arresting expansion due to elemental aluminum, and mortars made with that ash gave a high activity index, for some more than 100%. However, leaching of antimony and chromium from the mortars was observed to be above limits [26,52,83].

To increase the activity of ash as a pozzolanic material, its reaction can be activated through the addition of chemical activators. After adding MSWI bottom ash as a pozzolanic material, alkaline activators can be added to activate the reaction. It was found that CaCl_2_ is an effective activator, in contrast to Na-based activators, which did not improve the strength [28]. However, chloride in concrete can increase the susceptibility to reinforcement corrosion. Krammart and Tangtermsirikul used Ca(OH)_2_ as an alkali activator, and a calcium aluminate monosulphate (AFm) type phase, calcium aluminum carbonate sulfate hydrate (Ca_4_Al_2_O_6_(CO_3_)_0.67_(SO_3_)_0.33_·11H_2_O) and calcium silicate hydrate (CSH) with a low Ca-Si ratio were the hydration products formed. Since the material used was not treated to control the effect of aluminum, the compressive strength obtained was very low [32]. The use of bottom ash in a hybrid binder system consisting of OPC, MSWI bottom ash and fly ash, as well as a 5% mix of CaSO_4_ and Na_2_SO_4_ as the activator was investigated [54]. It showed a 40% strength loss for this mix in comparison with a pure OPC mix. The cement was found to immobilize the elements Pb, Zr and Sn. Nevertheless, the leaching of Cl^−^ was very high, raising the question of its usage in reinforced concrete. Gao et al. studied the use of washed fly ash as pozzolanic addition. Washing reduced chloride content from 10.16% down to 1.28%, and the addition of 0.25% dithiocarbamic chelate reduced heavy metal leaching. Ten percent replacement resulted in the same compressive strength as that of reference samples [125].

Another route for utilization of MSWI fly ash as pozzolanic material is by sintering ash, producing fly ash slag, and milling the slag further to produce a pozzolanic material. Lin et al. studied the hydration properties of C_3_S mixed with MSWI fly ash slag. A lower hydration degree of C_3_S-slag pastes compared to pure C_3_S pastes at all ages of hydration was observed. A pozzolanic reaction was observed after 28 days [126]. The effect of the addition of alumina prior to sintering was studied by Lin et al., who observed an increase in the degree of hydration up to 28 days for milled 10% alumina mixed slag blended cement pastes [127]. Similar research was carried out by Wang et al., which reported a reduction in early strength and an increase in 28-day strength due to 10–20% replacement of cement by milled fly ash slag [128]. Lin reported an increase in initial and final setting times for 10–40% cement replacement by municipal solid waste incineration fly ash and slag, possibly due to the presence of heavy metals like Zn, Pb and Cu [51]. It was also reported by Lee et al. that the early strength of mortars made with cement replaced with fly ash slag can be enhanced by increasing the basicity of slag by adding CaCO_3_ prior to sintering. Mortar with 20% cement replacement with fly ash slag with 20% CaCO_3_ additive had the same 14-day and 28-day compressive strength as that of reference OPC samples and roughly around an 8% increase compared to the reference at 90 days [129]. Lee et al. studied the effect of 20% cement replacement by vitrified slag made from fly ash and scrubber ash mix proportioned 1:3, with waste glass frit added to adjust the basicity of the mix. Leaching values of slags were well below the threshold limits, and the compressive strengths of mortars at 56 and 90 days were 5–10% more than those of the reference OPC mixes [130,131]. Lin et al. manufactured vitrified slag from a 50–50 mix of 1:3 fly ash scrubber ash mix and sludge from LED manufacturing. The ground slag, which proved to be safe in terms of leaching, was used to replace cement up to 30%, and the resulting mortars had a compressive strength 6–36% higher than that of the OPC reference [132].

#### 8.2.2. Autoclaved Aerated Concrete 

Autoclaved aerated concrete, also known as AAC, is industrially produced as aerated precast elements. Accelerated curing is done by autoclaving in high pressure steam, and this ensures higher strength development. Different technologies are prevalent today for aeration of concrete, and adding aluminum powder is one among them. MSWI ash inherently contains metallic aluminum, and thus, it can be an added advantage to be used as a raw material for AAC production. Furthermore, the aeration agent cost can be saved. Song et al. studied the effect of replacing fly ash or silica by incineration bottom ash. They reported an increase in compressive strength and a decrease in drying shrinkage with the increase in bottom ash replacement [46]. 

#### 8.2.3. Manufacture of Ceramics

The high content of silica in the filter dust makes it suitable for the production of glass ceramics. There are already successful attempts in utilizing fly ash and slag for that purpose. High temperature sintering of the ash generates high density crystalline products that can be used as a building material, and at the same time, it will bind hazardous inorganic materials, making it environmentally safer. Glass ceramics were manufactured by heating at a rate of 10 °C/min up to 880 °C, maintaining this temperature for 4 h and then heating again at 5 °C/min to 950 °C and maintaining it for 10 h, then later cooled down [133]. Pyroxene group minerals, mainly diopside, were the main crystalline products formed by sintering. The density of glass ceramic formed was 2.89 g/cc. A fracture mirror study yielded a fracture strength of 240 MPa. The chemical composition and microstructure of ceramic produced by milling, compacting and sintering of bottom ash below 8 mm in size were also studied [33]. Quartz, calcite, gehlenite and hematite were the main crystalline minerals in ash before sintering. Diopside was the main mineral formed as a result of sintering to an optimum temperature of 1080 °C and controlled crystallization along with clinoenstatite, wollastonite and albite. The maximum density obtained was 2.6 g/cc.

## 9. Cement Industry and Utilization Potential of MSWI Ashes in Belgium

Utilization of bottom ash in the cement industry is a promising solution technically, as we have seen already. The cement industry already recycles many waste streams as alternate raw materials. Furthermore, cement being the most used building material is consumed in huge quantities. It can be seen from Table 6 and Table 7 that around 2 MT of the cements CEM I, II and V were consumed in Belgium in 2015. Unutilized bottom ashes are available in 15% of this quantity by mass (~300 kT) [134].

**Figure 7:**
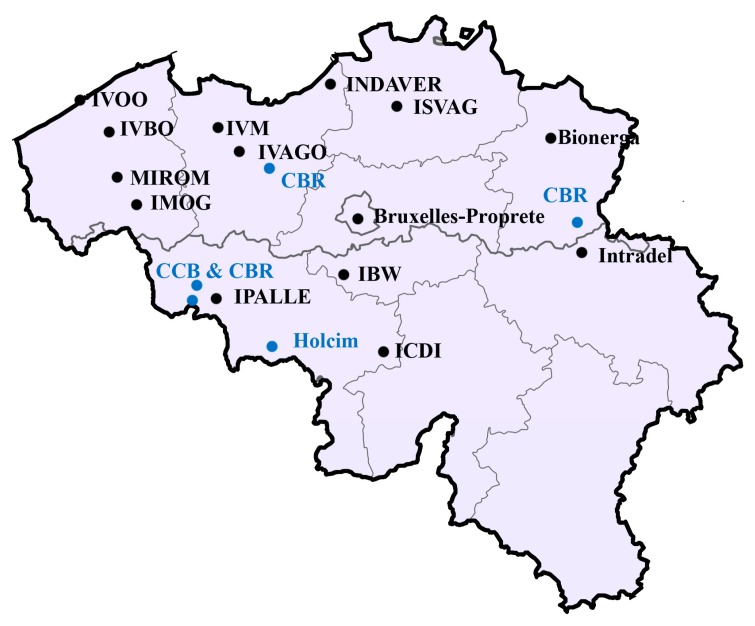
Areal distribution of major incineration plants and cement plants in Belgium (black, incineration plants; blue, cement plants) cf. [22,134].

At the same time, there is consumption of the CEM II type of cement that utilizes fly ashes from coal-fired thermal power plants. Since there are no longer thermal power plants in Belgium as of 2016, fly ashes for CEM II production need to be imported from neighboring countries. Transportation adds to the cost, energy consumption and CO_2_ emissions considerably and thus affects the portfolio of cements produced. Incineration plants have good areal distribution and thus save transportation costs, and treated bottom ashes could act as a replacement for fly ash in CEM II, as can be seen from the map in Figure 7. Therefore, the use as pozzolanic material or as a raw meal for clinker production are promising options for utilization of more bottom ashes. The <2 mm fraction of bottom ash is mostly unutilized, due to more heavy metals. Another major obstacle to the utilization is the presence of metallic aluminum. There are a number of techniques available to stabilize heavy metals such as ageing, wet grinding, treatment with sulfide-rich effluent, binder stabilization, vitrification, etc. Ageing of samples is already done in the field. Vitrification/slagging processes are both capital and energy intensive. Both wet grinding and treatment with sulfide-rich effluent could be possible options. Wet grinding can solve both obstacles. Metallic aluminum can be hydrated to aluminum hydroxide during the process, and it can be accelerated by utilizing waste heat generated in the plant [103]. Sources of waste heat from the plant include heat from the stack gas and heat generated during ageing that raises the temperature of bottom ash to 70–80 °C. It would be a challenging job for an industrial engineer to harvest this waste heat, but if realized, it can make the process more economic and sustainable. The second option is to use the ashes as a cement raw material. A part of the heavy metals, especially Cu, Zn and Pb, can be incorporated in the phases in cement; however, if above certain limits, it can alter them. The presence of metallic aluminum is not known to affect the production process. Relatively high alkali content in bottom ashes can affect the clinkerization process, thus limiting the replacement ratios to a smaller value. Nevertheless, even small replacement ratios can result in huge utilization of material due to the enormous quantities of cement produced. The next step for more utilization is testing these options in pilot scale in cement/incineration plants and optimizing the process. This will allow full utilization of bottom ash in a more high value product, i.e., cement, than using it as aggregate or as road subbase material, making it more economical. It helps to reach the goal of the circular economy utilizing many waste streams. Various research works for realizing the utilization of fly ashes are available. Many studies reported pozzolanic activity of fly ash slag made by vitrification of fly ashes, thus reducing its leaching. Being a capital- and energy-intensive technique, it might be more challenging to optimize the process. More research to identify the advantages of materials and using them for the right application will be the key to achieving a circular economy for fly ashes. 

## 10. Conclusions

This article discussed the utilization potential of MSWI ashes in a variety of fields, various obstacles preventing utilization and various options for treatment procedures that could overcome these obstacles. The composition of ash depends on the input waste, type of incinerator and process parameters, and this varies spatially and temporally; it does not hinder its utilization in one of the various potential fields of application. The most suitable area of utilization for each type of ash produced in around 2200 waste to energy plants around the world needs to be identified and put into use locally. Moreover, the cement industry, which is identified as the most suitable agent for valorizing bottom ashes as raw meal additive and supplementary cementitious material, is equipped for dealing with variability in raw material composition and still produces a final product with the required quality control. One of the sources of variation is the process parameters of incineration plants, which are optimized currently for maximum energy generation. However, the process parameters can have an effect on the chemical properties of final residues, which could in turn affect the technical quality of the final product after utilization. The process parameters of incineration plants need to be optimized for maximum utilization of residues. A comprehensive database of the effects of various characteristics of residues on the quality of final products after incineration needs to be developed after research. 

In Belgium, we have already come a long way, and pretreatment installations solve many problems to an extent. Evidence of the successful application of pre-treatment techniques at the laboratory scale is available. The most suitable options for higher value applications are usage as a binder or raw meal substitute. Use as cement raw meal additive requires less pre-treatment and thus is another promising option for high value application. The present need is to optimize the whole process flow technically and economically by conducting pilot scale tests and doing plant-specific life cycle analysis to identify what is gained and what not. Use as a pozzolanic additive is a good option for Belgium considering its almost zero production of coal fly ashes. Sequencing and combining the right pre-treatment techniques will be the key to the most sustainable utilization. The Revasol technique modified by Aubert et al. is one such method addressing various obstacles for use as pozzolana. New research for localized solutions for treatment of ashes with minimal secondary pollutions needs to be done. In Belgium, Cu and Zn content is the major obstacle preventing utilization of 164 kT of bottom ashes. Another major problem is the metallic aluminum content preventing its effective use as a pozzolanic additive and aggregate in concrete. Wet grinding of the ash at a higher temperature can be a solution for both of these, and the process can be optimized by using local waste water preferably containing sulfides and thus reducing the impact of secondary pollution of water. Hydrogen gas generated by washing can be harvested and used for energy generation. Another solution can be carbonation of ash, for which the high CO_2_ concentration of stack gas can be utilized or sequestrated. This can add to the economy of the plant, as well, due to the additional income from residue recycling. Capital-intensive techniques, like vitrification, which could be more effective for MSWI fly ash, can be employed after balancing the cost and benefit. Establishing the business model and development of standards and legislation are the key to a better recycling. Before that, comprehensive methodologies for utilization with emphasis on technical and environmental specifications need to be developed by the scientific community.

## Figures and Tables

**Table 1:** Nomenclature of waste streams from MSWI ash [8,24].

Name	Point of Collection
Grate ash	Ash collected from the grate
Grate siftings	Material collected from the hoppers underneath the grate
Bottom ash	Combined grate ash and grate siftings and sometimes heat recovery ash; it is mainly composed of bottle glass, metals, ceramics and organic residues [25]
Heat recovery system ash (HRA)	Ash collected from boiler, economizer and super heater
Fly ash	Raw particulate matter entrained in the flue gas stream prior to addition of scrubbing reagents. It is a type of Air Pollution Control residue
Air pollution control (APC) residue	All particulate material captured downstream of any reagent injection and prior to discharge of gases to stack; its reuse will be more difficult due to the significant presence of heavy metals and toxic compounds like Polychlorinated dibenzo-p-dioxins (PCDDs) and polychlorinated dibenzofurans (PCDFs) [26]
Combined ash	Mixture of bottom ash, grate siftings and APC residues
Sintered ash	Bottom ash or fly ash is sometimes sintered and solidified, thus reducing the leaching and facilitating utilization

**Table 2:** Mineralogy of bottom ashes, fly ashes and sintered ashes from MSWI ash.

Minerals Identified	Reference	Occurrence and Potential Use
Quartz (SiO_2_)	[26,33,34,35,49,51]	It acts as an inert filler when used in cement as SCM. Furthermore, it can have pozzolanic properties when very finely ground. It can be a source of silica when used as a cement raw material.
Calcite (CaCO_3_)	[33,34,35]	It can contribute carbonate to the system, leading to stabilization of ettringite and mono-carbonate/hemi-carbonate when used as an SCM, depending on the content of C_3_A. The rest of the calcite will act as a filler. Calcite is the commonly-used source of calcium, thus highly beneficial for clinker production.
Gehlenite (Ca_2_Al_2_SiO_7_)	[26,33,35,51]	Inert constituent in calcium aluminate cements, carbonatable.
Hematite (Fe_2_O_3_)	[26,33,34]	Largely inert, formed during incineration.
Magnetite	[25,49]	High temperature phase/inert.
Ettringite	[49]	Mainly formed by quenching of bottom ash, from the reaction between sulfates and reactive aluminates.
Hydrocalumite	[49]	Mainly formed by quenching of bottom ash.
Diopside (CaMgSi_2_O_6_)	[33]	Principal crystalline phase of sintered ash.
Clinoenstatite (MgSiO_3_)	[33]	Found in sintered ash/ceramics.
Wollastonite (CaSiO_3_)	[33]	Found in sintered ash/ceramics.
Ingersonite (γ-Ca_2_SiO_4_)	[50]	Reactive towards CO_2_.
Hedenbergite	[25]	Slag/ash component-inert.
Ferrohedenbergite	[25]	Slag/ash component-inert.
Feldspar	[25]	Common inert rock-forming mineral.
Melilite (Ca,Na)_2_(Al,Mg,Fe^2+^)[(Al,Si)SiO^7^]	[25,49]	Contains Mg; carbonatable.
Albite (NaAlSi_3_O_8_)	[33,34]	Found in sintered ash/ceramics.
Anorthite (CaAl_2_Si_2_O_8_)	[35,47,51]	Common inert rock-forming mineral.
Anhydrite (CaSO_4_)	[26,34,51]	Cement constituent, added to control setting.
Gypsum (CaSO_4_·2H_2_O)	[36,49]	Cement constituent, added to control setting.
Gismondine (CaAl_2_Si_2_O_8_·4H_2_O)	[50]	
Apatite(Ca5(PO_4_)_3_(OH,F,Cl))	[26]	Fly ash treated by washing, phosphation and calcination to 750 °C. Bone fragments can also be a source of apatite in ash.
Whitlockite (β-Ca_3_(PO_4_)_2_)	[26]	Fly ash treated by washing, phosphation and calcination to 750 °C.
Titanite (CaTiSiO_5_)	[26]	Fly ash treated by washing, phosphation and calcination to 750 °C.
Perovskite (CaTiO_3_)	[26,52]	Inert.
Periclase	[25]	Carbonatable.

**Table 3:** Estimation of hydrogen gas production.

Aluminum in Ash (%)	Theoretical Volume of Hydrogen Produced in 1 m^3^ of Concrete (Assuming 25% Replacement of Cement by Ash and 450 kg/m^3^ of Cement Content) in m^3^; cf. Equation (2) at STP (Standard Temperature and Pressure)
0.1	0.150259
1	1.502592

**Table 4:** Comparison of Flemish and Wallonia guidelines and typical concentrations in bottom ash cf. [62,63].

Parameter	Flanders Criteria (VLAREMA) Total Concentration Limit mg/kg Dry Matter	Wallonia Criteria Total Concentration Limit mg/kg Dry Matter	Bottom Ash Total Typical Concentration mg/kg Dry Matter
Arsenic	250	100	33 ± 17
Cadmium	10	8	
Chromium	1250	230	482 ± 73
Copper	375	210	4042 ± 888
Mercury	5	15	3 ± 2
Lead	1250	1150	1899 ± 396
Nickel	250	150	329 ± 69
Zinc	1250	680	5376 ± 782

**Table 5:** Summary table.

Obstacle	Pre-Treatment Technique	Advantages	Disadvantages
Metallic Aluminum and Zinc	Magnetic density separation	Versatile Simple	Initial cost, not for fines
	Eddy current separation	Can detect through several layers Can provide an accurate separation	Initial cost, not for fines Susceptible to magnetic permeability changes
	Wet grinding	Consumes lower power per ton of product. Enables the use of wet screening or classification for close product control. Eliminates the dust problem. Enables the use of simple handling and transport methods such as pumps, pipes and launders.	Storage of wet slurries
	Washing with alkali	Simple	Cost of alkali
Salts	Washing with water	Simple	Secondary pollution of water, unless the water in the slurry is used to make concrete from the material
	Carbonation	CO_2_ from stack gas can be utilized and thus reduce the emission. Can be a method for carbon sequestration.	Not a very fast process, unless the CO_2_ concentration is very high, which in turn will require air tight enclosures
	Thermal treatment	Simple technology	Energy Consumption Cost
Heavy metals	Washing with water	Simple	Secondary pollution of water
	Treatment with sulfide rich effluent	Simple and can utilize another waste stream	Applicable for specific heavy metals
	Wet grinding	Simple No addition of additional chemicals	Storage of wet slurries
	Phosphation	Stabilizes heavy metals	Applicable only for specific applications
	Cement stabilization	Stabilizes heavy metals	Cost of cement
	Hydrothermal treatment		Capital cost
	Electrodialytic remediation		Costly Energy Consumption

**Table 6:** Cement consumption in Belgium cf. [134].

Categories	Cement Consumption in 2015 (MT)
Concrete products and fiber cement	1
Ready mix concrete	2.767
Delivery in construction site	0.743
Delivery in hardware shops	0.381
Total consumption	4.891
Import	1.513
Export	1.384

**Table 7:** Percentage consumption of various classes of cement in Belgium in 2015 cf. [134].

Cement Type	Strength Class	% Consumption
CEM I, II, V	32.5	12
	42.5	3
	52.5	26
CEM III	32.5	8
	42.5/52.5	51
